# Sensitive and Rapid Detection of Citrus Scab Using an RPA-CRISPR/Cas12a System Combined with a Lateral Flow Assay

**DOI:** 10.3390/plants10102132

**Published:** 2021-10-08

**Authors:** Kihye Shin, Soon-Hwa Kwon, Seong-Chan Lee, Young-Eel Moon

**Affiliations:** Citrus Research Institute, National Institute of Horticultural and Herbal Science, Rural Development Administration, Jeju 63607, Korea; shkwonn@korea.kr (S.-H.K.); scjmbr@korea.kr (S.-C.L.); yimoon@korea.kr (Y.-E.M.)

**Keywords:** citrus scab, *Elsinoë fawcettii*, recombinase polymerase amplification, CRISPR/Cas12a, lateral flow assay

## Abstract

Citrus is the most extensively produced fruit tree crop in the world and is grown in over 130 countries. Fungal diseases in citrus can cause significant losses in yield and quality. An accurate diagnosis is critical for determining the best management practices and preventing future losses. In this study, a Recombinase polymerase amplification (RPA)-clustered regularly interspaced short palindromic repeats (CRISPR)/associated (Cas) system was established with the integration of a lateral flow assay (LFA) readout system for diagnosis of citrus scab. This detection can be completed within 1 h, is highly sensitive and prevents cross-reactions with other common fungal citrus diseases. Furthermore, the detection system is compatible with crude DNA extracted from infected plant tissue. This RPA-CRISPR/Cas12a-LFA system provides a sensitive, rapid, and cost-effective method with promising and significant practical value for point-of-care diagnosis of citrus scab. To our knowledge, this is the first report to establish an RPA- and CRISPR-based method with LFA for fungal diseases in plants.

## 1. Introduction

Citrus scab is caused by the fungal pathogen *Elsinoë fawcettii* Bitancourt and Jenkins. This fungal disease affects young leaves, twigs, and fruit, and it has serious effects on the quality of the fruit, which reduces its market value [1,2]. The typical symptoms of citrus scab are a pale-yellow pustule with a corky mass on the infection site [3,4].

Plant fungal diseases are diagnosed via observation of symptoms through field inspections and laboratory-based tests, such as cultivation of pathogens on agar media, followed by physiological, biochemical, and pathogenicity tests, however, these methods are time-consuming and require skills and expertise. For these reasons, the development of a new, adaptable, and deployable plant disease diagnosis method is required [5].

DNA-based approaches are effective and accurate tools for diagnosing plant diseases. These methods are based on the amplification of the DNA of species-specific genes using polymerase chain reaction (PCR), quantitative real-time PCR, and loop-mediated isothermal amplification (LAMP) [6]. To diagnose citrus scab, PCR methods (including real-time PCR) are widely used [7,8,9], however, these methods also require trained skills and expensive equipment, which limits their application in the field and in resource-limited situations.

Recombinase polymerase amplification (RPA) is an isothermal DNA amplification technology that is based on three enzymes: a strand displacing DNA polymerase, a DNA recombinase, and single strand binding proteins (SSBs). Briefly, a DNA recombinase binds to primers and forms a DNA recombinase-primer complex to recognize the targeted DNA template. Next, the SSBs binds simultaneously to the displaced strands of DNA and stabilizes the formed nucleoprotein filament. Then, a strand displacing DNA polymerase recognizes the bound complex and initiates DNA synthesis [10,11,12]. RPA is a highly sensitive and rapid amplification method that has been used in a wide range of applications that require DNA amplification, including disease diagnosis, transgene detection, and food safety tests [13,14,15]. RPA assays can be performed within 20 min at a constant temperature ranging from 25 to 45 °C. Other advantages of RPA are that it is tolerant of numerous substances that inhibit amplification in PCR-based assays, and the reagents are sold in a lyophilized format suitable for in-field application [10]. This allows for a simple and rapid diagnosis of plant diseases.

Recently, novel diagnostic methods based on clustered regularly interspaced short palindromic repeats (CRISPR)/associated (Cas) systems have provided a promising approach for rapid and accurate detection. DNA-endonuclease-targeted CRISPR trans reporter (DETECTR) has been used for Cas12a-assisted nucleic acid detection. Cas12a is an RNA-guided nuclease that cleaves double-stranded DNA containing a T-rich protospacer-adjacent motif (PAM). After cleavage, Cas12a cleaves the surrounding single-stranded DNA (ssDNA), which is also called reporter DNA, in a nonspecific manner. Combined with RPA, DETECTR has successfully been used to perform highly sensitive and specific detection of various pathogens [16,17,18,19,20].

Additionally, easy visualization of the RPA-CRISPR/Cas12a results using lateral flow devices can be attained without the involvement of fluorescence readers, electrophoresis, and a source of ultraviolet radiation. The lateral flow assay (LFA) is an emerging detection device that can be read with the naked eye to detect amplified DNA products. In the classical LFA, amplified DNA with a Fluorescein phosphoramidite (FAM)/Fluorescein isothiocyanate (FITC) at one end and biotin at the other end is captured by biotin-binding protein (streptavidin) in the bottom line, which is close to the sample pad. Then, the FAM/FITC are recognized and bound by gold conjugate nanoparticle (Au-NP)-labelled anti-FAM/FITC antibodies. Therefore, the bottom line generates a color signal. In the CRISPR/Cas-based detection system, double-labelled single-strand DNA (ssDNA) is used as reporter and a defined amount of reporter is used in the LFA. Without target amplicon, the intact reporters are captured, and a majority of the gold conjugate is trapped in the bottom line, which leads to a weak signal in the top line referring a negative result. In the presence of target amplicon, the reporters are cleaved, and the labels are separated from each other by activated Cas protein. Consequently, less of the mobile gold conjugate can be retained on the bottom line. The bottom line intensity decreases and the top line intensity increases which refers positive result [21,22,23,24].

A number of CRISPR/Cas12a diagnosis assays have been developed for the rapid detection of plant pathogens, such as potato virus X; potato virus Y; tobacco mosaic virus [25]; tomato mosaic virus; tomato brown rugose fruit virus [26]; tomato yellow leaf curl virus; tomato leaf curl New Delhi virus [27]; and beet necrotic yellow vein virus [28]. Recently, in citrus, RPA-CRISPR/Cas12a detecting method for *Candidatus* Liberibacter asiaticus, which is the causal agent of citrus greening—Huanglongbing, was developed [29], however, CRISPR/Cas12a diagnosis assays for fungal plant pathogens have not yet been developed, even though such pathogens are easily widely spread and require early diagnosis for efficient control.

**Figure 1:**
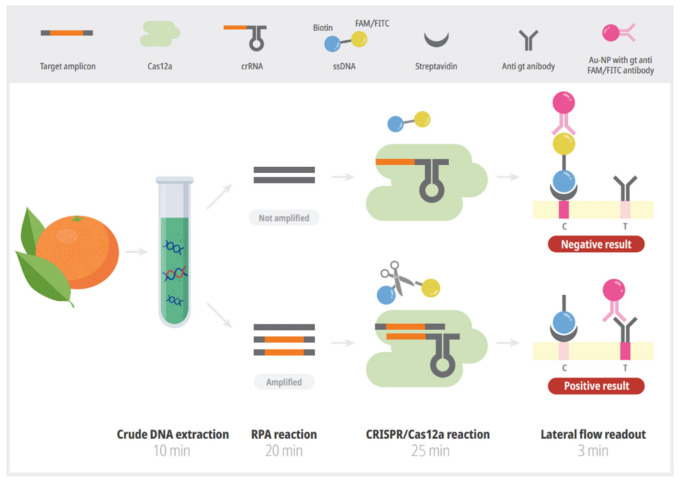
Schematic of the simple and rapid citrus scab detection process using the RPA-CRISPR/Cas12a-LFA assay. Unique genomic regions of *Elsinoë fawcettii* were amplified in an RPA reaction in 20 min at 37 °C, using crude DNA extract. The amplification product was added into a CRISPR/Cas12a mixture, and a trans-cleavage reaction was activated in the presence of the target amplicon and was completed in 25 min at 37 °C. The result was visible in 3 min using lateral flow readout. Without target amplicon, the intact ssDNA is captured in C line and then gold nanoparticle is trapped in the C line which refers negative result. With target amplicon, the labels are separated from each other by activated Cas12a and gold nanoparticle is trapped in the T line which refers positive result. ssDNA: single stranded DNA, streptavidin: biotin-binding protein, gt: goat, Au-NP: gold conjugated nanoparticle, C: control line, T: test line.

In this study, we developed a rapid and sensitive RPA-CRISPR/Cas12a-LFA assay to detect the causal agent of citrus scab, *E. fawcettii*. It was possible to use a crude DNA extract from infected citrus leaves and fruit as template for the RPA reaction. After 20 min of RPA reaction, the amplicon was added to the CRISPR/Cas12a mixture and incubated for 25 min. Then, the detection results were visualized through LFA (Figure 1).

## 2. Materials and Methods

### 2.1. Preparation of Positive Control

*E. fawcettii*, SM16-1 isolate, a Florida broad host range pathotype, which was deposited at the Korea Agricultural Culture Collection (KACC45780) was used in this study. Dried and preserved mycelia of *E. fawcettii* were cut into small pieces and cultured on potato dextrose agar (PDA) (Becton, Dickinson and Company, East Rutherford, NJ, USA) at 25 °C for 2 weeks. Total DNA was extracted with the Bioneer AccuPrep^®^ Genomic DNA Extraction Kit (Seoul, Korea) and used as template for RPA reaction and for conventional PCR as positive control.

### 2.2. RPA Reaction

The species-specific sequences of internal transcribed spacers (IST) 1 and 2 between the 16S and 23S rRNA gene loci are used for identification of microbes at species and sub-species level. ITS1 and ITS2 from *E. fawcettii* were downloaded from NCBI and used as templates for the primer design, as previously reported [7]. Several species-specific candidates forward (F) and reverse (R) primer sets (Table 1) were manually designed according to the TwistAmp Manufacturer’s Principles Kit (TwistDX, Cambridge, UK). The primers were synthesized by Bioneer (Seoul, Korea).

The RPA reactions were established according to the instructions in the TwistAmp Basic Kit (TwistDX) with few modifications. Briefly, a mixture of 2 μL of template DNA, 1 μL of forward primer, 1 μL of reverse primer, and 29.5 μL of rehydration buffer (TwistDX) was finalized to 47.5 μL with double-distilled water, and 2.5 μL of magnesium acetate (MgOAc) was added before starting the reaction. The reactions were performed at 37 °C for 20 min.

### 2.3. CRISPR/Cas12a-LFA Detection

The sequence of the guide RNAs that recognized the specific sequences of the RPA amplicons is listed in Table 1 (denoted as crRNA). For the lateral flow detection of the Cas12a-mediated trans-cleavage of the ssDNA reporter, the ssDNA was labeled at 5′ with biotin and at 3′ with FAM (5′-/6-FAM/TTATT/biotin/-3′). The crRNA and ssDNA were synthesized by Bioneer (Seoul, Korea). The EnGen Lba Cas12a enzyme (NEW ENGLAND BioLabs, NEB, Ipswich, MA, USA) was preincubated at a concentration of 1 μM with 1 μM crRNA in 1× NEBuffer 2.1 (NEB) at 37 °C for 10 min to form a ribonucleoprotein (RNP) complex. A reaction mixture of 20 μL of Cas12a containing 1 μL of Lba Cas12a (1 μM), 2 μL of NEBuffer 2.1 (NEB), 1 μL of crRNA (1 μM), 2 μL of RPA products, 1 μL of the FAM–biotin-labeled probe (10 μM), and 13 μL of water was incubated at 37 °C for 30 min. After incubation, 20 μL of the reaction mixture was mixed with 100 μL of HybriDetect assay buffer (Milenia Biotec, Giessen, Germany) in a 1.5 mL tube. A lateral flow strip (Milenia Biotec) was placed upright in the tube at room temperature (usually, bands appear on it within 2–3 min).

### 2.4. Sensitivity and Specificity of RPA/Cas12a-LFA Detection

The amplification sensitivity of the RPA/Cas12a-LFA detection of citrus scab was compared with that of conventional PCR using electrophoresis on a 2% agarose gel. The amplification sensitivity limit was determined by using 1:10 serially diluted genomic DNA of *E. fawcettii* down to 1 fg (1, 10^−1^, 10^−2^, 10^−3^, 10^−4^, 10^−5^, 10^−6^ ng).

To evaluate the specificity of the RPA/Cas12a-LFA detection, gDNA was extracted from 5 different causal agents of fungal citrus diseases—*Diaporthe citri*, *Glomerella cingulata*, *Alternaria citri*, *Fusarium* spp., and *Botrytis cinerea*—and was used as a DNA template. Distilled water was used in the negative control reaction. The experiments were repeated three times.

### 2.5. Preparation of Crude Extract of Plant Samples

To test the on-field applicability, infected citrus leaf tissues were collected, and total DNA was extracted with a simple and crude method. One gram of scab-infected tissue and one gram of healthy tissue placed in 2 mL of TE buffer; 10 mM Tris-HCl, 0.1 mM EDTA, pH 7.6, Polyethylene glycol (PEG) buffer; 6% PEG 200 (Sigma-Aldrich, Gillingham, UK) with 20 mM NaOH, and GE3 buffer; general extraction 3 (Agdia, Elkhart, IN, USA) were ground using a mesh-lined bag (ACC 00930, Agdia, Elkhart, IN, USA) and a hand-rolling tissue homogenizer (ACC 00900, Agdia). For each reaction, 2 μL of crude extract was used as a template DNA.

### 2.6. PCR for Field Sample

Field samples were harvested from satsuma mandarin leaves and fruits suspected to be infected with scab. Total plant DNA was extracted using extraction methods of DNeasy Qiagen plant mini kit (Qiagen, Valencia, CA, USA) and used as PCR templates. For conventional PCR, *E. fawcettii* specific primers described in previous reports for the detection of citrus scab were used in PCR assay [7]. A commercial PCR premix (AccuPower PCR PreMix, BIONEER, Daejeon, Korea) was used according to the manufacturer’s recommended protocol. Briefly, a mixture of 1 μL of template DNA, 1 μL of each primer (10 μM), and 17 μL of distilled water was added to a 0.2 mL PCR tube that contained a pelleted enzyme and buffer. PCR cycling conditions were 5 min at 95 °C followed by 35 cycles of 30 s at 95 °C, 30 s at 64 °C, and 30 s at 72 °C, and 10 min at 72 °C using thermal cycler (Biorad C1000 cycler, Hercules, CA, USA).

## 3. Results

### 3.1. Screening of Primers for the RPA Reaction

**Figure 2:**
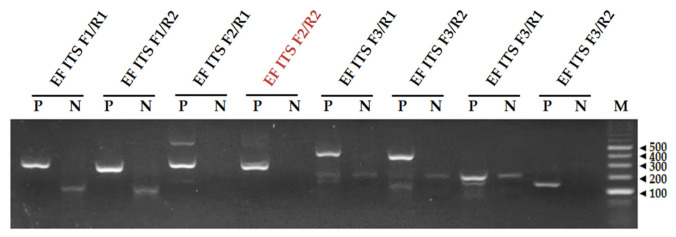
Screening of primer pairs with an RPA assay. The names of the primer pairs are indicated on the top. The sizes of the DNA ladder bands are shown on the right (bp). EF ITS: *Elsinoë fawcettii* internal transcribed spacers, F: forward primer, R: reverse primer, P: presence of template, N: no template, negative control, M: size marker. The reactions were performed at 37 °C for 20 min.

The primer pairs were screened for their amplification performance in the RPA reactions using electrophoresis; the sizes of the amplicons were distinguishable (Figure 2). The total DNA of *E. fawcettii* was used as a template for the RPA reaction. The *E. fawcettii* RPA reaction results showed that most of the primer pairs gave clear specific bands, but some pairs also showed nonspecific bands (EF ITS F2/R1). In the no-template sample (N), some pairs formed primer dimers bands. Because of its specificity and lack of primer dimer formation, the EF ITS F2/R2 primer pair was selected as a candidate for the RPA detection of *E. fawcettii* (Figure 2).

### 3.2. Establishment of RPA-CRISPR/Cas12a-LFA Detection

The principle of the RPA-CRISPR/Cas12a-LFA assay is illustrated in Figure 1. The unique region of *E. fawcettii* was amplified through an RPA reaction, and the reaction product was directly added to the CRISPR/Cas12a reaction. The amplicon with a unique sequence could be specifically recognized by Cas12a and subsequently triggered CRISPR/Cas12a to cleave the double-labeled ssDNA reporter through the trans-cleavage activity [19,24]. The lateral flow readout is based on the cleavage of a double-labeled reporter (FAM/FITC–biotin); the presence of many reporters causes anti-FAM antibody–gold nanoparticle conjugates (Au-NP) to gather at the C (control) line on the strip. In our experiments, when the activated CRISPR/Cas12a cleaved the ssDNA FAM/FITC–biotin reporter, the band intensity significantly increased on the T (test) line and decreased on the C line [30] (Figure 1). We optimized the concentration of the ssDNA reporter (data not shown) and chose 10 μM.

**Figure 3:**
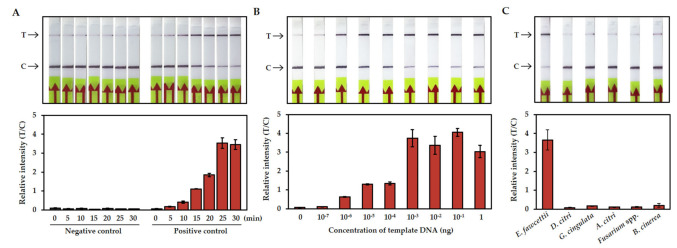
The limit of detection with RPA-CRISPR/Cas12a-LFA. (**A**) Optimal incubation time of the CRISPR/Cas12a reaction. The LFA strip results for different reaction times are shown at the top, and the relative quantifications of band intensities are shown below. NC: negative control, no template, PC: positive control with 1 ng of the total gDNA of *E. fawcettii*, T: test line, C: control line. (**B**) The sensitivity of the RPA-CRISPR/Cas12a-LFA detection was determined using ten-fold serially diluted gDNA as a template. (**C**) The specificity of the RPA-CRISPR/Cas12a-LFA detection was tested with the causal agents of five common fungal citrus diseases. All the experiments were performed three times, and representative pictures are shown. The error bars indicate the standard deviations.

The optimal incubation time of the CRISPR/Cas12a reaction was tested with seven time points (0, 5, 10, 15, 20, 25, 30 min). In the positive control sample, the signal on the T line gradually increased and that on the C line gradually faded with the progression of the incubation time. The band intensity analysis showed that the ratio of the intensities of the test and control bands was greater than one after 15 min of incubation. After 25 min, the ratio was increased 30-fold compared with the negative control (no template) (Figure 3A). For a clear and obvious decision, we set the incubation time to 25 min for the standard protocol and further experiments.

### 3.3. Sensitivity and Specificity of the RPA-CRISPR/Cas12a-LFA Detection

The sensitivity of the RPA-CRISPR/Cas12a-LFA detection was determined by using serial dilutions that ranged from 1 ng to 1 ag of gDNA. The RPA reactions were performed as described above at 37 °C for 20 min, and the CRISPR/Cas12a reactions were incubated at 37 °C for 25 min. The LFA results showed the clearly increased signal of the T line at the limit of l0^−6^ ng (1 fg) of template DNA. When the template concentration was over 10^−3^ ng (1 pg), the signal of the T line was stronger than that of the C line, and the ratio of the intensities of the T and C bands was saturated (Figure 3B).

To evaluate the specificity of the detection, the gDNAs of causal agents of five common fungal citrus diseases were tested. *D. citri*, *G. cingulata*, *A. citri*, *Fusarium* spp., and *B. cinerea* are fungal pathogens that cause citrus melanose, anthracnose, Alternaria disease, dry root rot, and botrytis disease, respectively [31,32,33,34,35]. Figure 3C shows that only the reaction with *E. fawcettii* had a positive signal. Taken together with the sensitivity test, these results demonstrated that the RPA-CRISPR/Cas12a-LFA citrus scab detection system is highly sensitive and specific and does not have cross-reactions with non-scab fungal pathogens.

### 3.4. Citrus Scab Detection with Infected Field Samples

**Figure 4:**
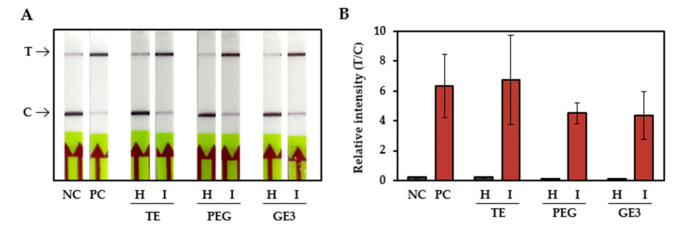
Sensitivity of the RPA-CRISPR/Cas12a-LFA system with crude DNA extracted from infected citrus leaf samples. DNA extracted from fungal pathogens was used as a positive control. (**A**) The LFA strip result for DNA of citrus-scab-infected leaves and healthy citrus leaves extracted with different extraction buffers. (**B**) The relative quantifications of band intensities. The experiments were performed three times, and representative pictures are shown. The error bars indicate the standard deviations. T: test line, C: control line, NC: negative control, no template, PC: positive control, pathogen DNA template, H: healthy sample, I: infected sample, TE: Tris-EDTA buffer, PEG: Polyethylene glycol, GE3: general extraction 3.

To simplify the steps for citrus scab detection, we tested the sensitivity of the RPA-CRISPR/Cas12a-LFA system with crudely extracted DNA samples. The DNA of citrus-scab-infected leaves and healthy citrus leaves was extracted with TE buffer, PEG buffer, and GE3 buffer. The infected leaf samples were placed inside a mesh-lined sample bag and squeezed with a hand-rolling tissue homogenizer. A total of 2 μL of tissue lysate was directly used for the RPA reactions followed by CRISPR/Cas12a-LFA. Figure 4 shows that all three samples, obtained with different extraction buffers, had positive signals. These results suggested that the lysates obtained using any of these three buffers can be successfully used as input material for the detection of citrus scab in the RPA-CRISPR/Cas12a-LFA system.

**Figure 5:**
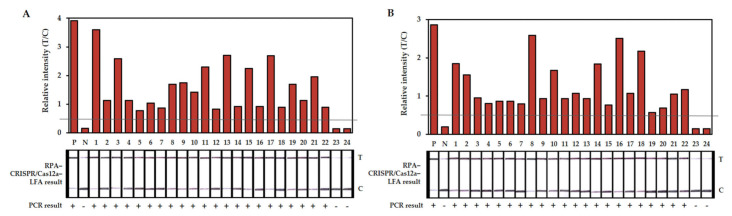
Scab diagnosis of field leaf samples (**A**) and fruit samples (**B**) using RPA-CRISPR/Cas12a-LFA and PCR. The relative quantifications of band intensities are shown at the top and LFA strip results are shown at the middle and PCR results are shown at the bottom. The gray horizontal line indicates the relative intensity threshold for positive result. N: negative control, no template, P: positive control with 1 ng of the total gDNA of *E. fawcettii*, T: test line, C: control line.

To validate and evaluate the feasibility of the RPA-CRISPR/Cas12a-LFA system, 44 different samples (22 leaf samples and 22 fruit samples) suspected to be infected with scab and 4 healthy samples (2 leaf samples and 2 fruit samples) were tested, and the results were compared with those of PCR detection (Figure 5). The RPA-CRISPR/Cas12a-LFA system and the PCR were used in parallel with tissue lysate and the purified DNA, respectively. The results showed that all the citrus-scab-positive samples confirmed by PCR were also confirmed by the RPA-CRISPR/Cas12a-LFA system, and all the citrus-scab-negative samples showed negative signals when tested with both methods (Table 2).

## 4. Discussion

Citrus is one of the most important fruit crops in the world, however, citrus diseases caused by fungal, bacterial, and viral pathogens affect the quality and yield of the fruit. Specifically, citrus fungal disease can spread within a few years and have huge economic impacts [36]. Several approaches, such as chemical pesticide treatments, biological control, and treatment with other synthetic molecules, have been used for the control of these diseases [37,38,39,40]. These disease control strategies should be established with consideration of the environmental conditions, the developmental stages of the trees, vulnerable points in the disease cycle, and the degree of the disease, etc. [41,42,43]. Therefore, accurate and rapid diagnosis of a disease is required to effective management of any disease.

Here, we developed a rapid and sensitive RPA-CRISPR/Cas12a-LFA system for diagnosing citrus scab. This method has several advantages compared to conventional PCR-based methods. First, the RPA and CRISPR/Cas12a reactions can be performed at constant and relatively low temperature (37 °C), therefore, eliminating the requirement of expensive special instruments such as thermocyclers. The reactions can be performed in closed hands or with a hand warmer. Second, the entire diagnostic process can be completed within approximately 60 min, including 10 min for crude DNA extraction, 20 min for the RPA reaction, 25 min for the CRISPR/Cas12a reaction, and 5 min for visual detection using a lateral flow assay strip. In contrast, PCR-based detection method requires at least 3 h including 60 min for DNA extraction, 90 min for PCR reaction, and 30 min for gel electrophoresis (Figure 1). Third, the result can be easily read out using LFA strips which is not require special technique and equipment such as electrophoresis. Last, it is not necessary to use high quality and purified DNA for amplification template which is required for conventional PCR-based diagnosis (Figure 4). Taken together, this newly developed technique has great potential for use as a point-of-care diagnostic tool. On-field diagnosis of citrus scab could help in creating an effective and accurate control strategy for farmers.

The sensitivity and specificity assays revealed that the RPA-CRISPR/Cas12a-LFA system detected a minimum amount of 1 fg of *E. fawcettii* gDNA without cross-reactions for non-scab fungal pathogens (Figure 3). The development of sensitive, specific, rapid and equipment-free citrus scab diagnostic method has great advantages in terms of providing potential to be used for large scale screening and, hence, would serve as a valuable tool for citrus breeding research and citrus industries.

Recently, automated image-based diagnosis or classification systems that use artificial intelligence have been widely developed, and these require sufficient image data [44,45,46], however, before collecting images, accurate diagnoses of diseases should be made; otherwise, ambiguous data can give false information. Using the RPA-CRISPR/Cas12a-LFA citrus scab detection system, accurate and high-throughput data collection is possible.

In conclusion, a novel rapid, sensitive, and equipment free citrus scab pathogen detection method was developed in this study. The RPA-CRISPR/Cas12a detection system is combined with LFA to visualize the detection result. Furthermore, this detection system could be applied to a crude DNA sample which makes the whole diagnosis process easier. This new diagnostic method has the potential to be used for point-of-care diagnosis, enabling early disease diagnosis in citrus orchards, allowing a correct control plan to be established, thereby increasing the productivity of fruit. Also, since this method can be used for mass diagnosis, it has the potential to be used in breeding research, quarantine, and machine learning for artificial intelligence diagnosis.

## Figures and Tables

**Table 1:** The primers used in the RPA reaction and the CRISPR guide RNA (crRNA) in the Cas12 reaction.

Name	Sequence (5′–3′)	Length (bp)
EF ITS F1	AACCAACTCTTGTCTTGTGAAACCTTTGCAGT	32
EF ITS F2	CCGGGGGACCGAACCAACTCTTGTCTTGTGAAA	33
EF ITS F3	ACTCCCCACCCTTTGCTGTTGCGAATCACGTTG	33
EF ITS R1	AATACCAAGCGGGGCTTGATTGGTGAAATGAC	32
EF ITS R2	GGGGCTTGATTGGTGAAATGACGCTCGAACAGG	33
crRNA	UAAUUUCUACUAAGUGUAGAUAACGCACAUGCGCCCCUUG	40

**Table 2:** Test of the specificity of the RPA-CRISPR/Cas12a-LFA system compared with that of conventional PCR analysis with field samples.

	Leaves		Fruits	
	PCR Positive	PCR Negative	PCR Positive	PCR Negative
RPA-CRISPR/Cas12a-LFA positive	22	0	22	0
RPA-CRISPR/Cas12a-LFA negative	0	2	0	2
	PPA ^1^ (44 of 44 = 100%)		NPA ^2^ (4 of 4 = 100%)	

^1^ PPA, positive predictive agreement. ^2^ NPA, negative predictive agreement.
